# Prevalence and factors associated with substance use among university students in South Africa: implications for prevention

**DOI:** 10.1186/s40359-022-00987-2

**Published:** 2022-12-15

**Authors:** Stacey Blows, Serena Isaacs

**Affiliations:** Department of Psychology, Faculty of Community and Health Sciences, University of the Western Cape, Western Cape, South Africa

**Keywords:** College, Prevalence, Substance use, University, Young adults, Mental health

## Abstract

**Background:**

Substance use is an important public health concern in many countries across the globe. Among the general public, institutions of higher learning have developed a reputation for inducing new substance use among students. In addition to socio-demographic factors, substance use and abuse among university students often appear to be related to psychological stressors typically related to the demand to adapt to the new environment and the pressures associated with academia. The purpose of this study was to identify the prevalence of, and factors associated with substance use among university students.

**Methods:**

This quantitative study employed convenience sampling to recruit university students who were 18 years and older from a university in the Western Cape. The study made use of self-administered online questionnaires, which participants completed via SurveyMonkey. The sample consisted of 2915 students. Descriptive statistics were used to describe and provide the prevalence and overview of the demographic characteristics of the respondents. Associations between variables were explored using Chi-square and Mann–Whitney U tests.

**Results:**

The main findings revealed a substance use prevalence rate of 62.7%. The most prominent substances used by students were alcohol (80.6%), cannabis (46%), and ecstasy (5.3%). The study further  revealed clear associations between students’ substance use and mental health. Students who reported substance use at university reported higher depression and anxiety scores than those who did not. However, findings reveal no significant association (*p* = 0.233) between being a substance user and a nonsubstance user and students' respective self-perceived stress scores.

**Conclusion:**

Results show the majority of sampled students had started using substances (both alcohol and other substances) only after entering university. The results call into question seminal findings relating to substance use and the university environment. The novel findings of this study could serve as a baseline input to inform policymakers, programme developers, service providers, parents, and other stakeholders who are involved in the design and implementation of more effective awareness, prevention and, needs-based intervention services.

**Supplementary Information:**

The online version contains supplementary material available at 10.1186/s40359-022-00987-2.

## Background

Substance use is an important public health concern in many countries across the globe. Among the general public, institutions of higher learning have developed a reputation for inducing new substance use among students [1–3]. In addition to reporting novice use, studies have also found that students who had prior exposure to substance use increased their frequency once exposed to the university environment [4, 5]. A growing body of research has also shown that university students reported using a number of substances at a greater rate than their non-student peers [1, 6–9]. Findings of such studies show that the use of alcohol, particularly getting drunk and binge drinking [1, 6], marijuana [1] and non-prescription amphetamine, were considerably higher among university students when compared with their non-university attending peers [1, 7–9].

Research suggests that there could exist some conditions within the environment of higher education settings that makes students more susceptible to the use and/or abuse substances [4, 10–12]. The term "substance use" refers to the use of alcohol, tobacco, illicit drugs, prescription and over-the-counter medications [13]. “Substance abuse” refers to the continued misuse of drugs, alcohol, tobacco and other psychoactive drugs even though the individual has knowledge that their usage of these substances may cause several debilitating problems and may eventually lead to some form of addiction [14].

Although much is known about students’ substance use rates on a global scale, very few representative studies have been conducted in South Africa (see e.g., [15–19]). Among the few studies carried out in South Africa, very high rates of student alcohol use have been reported [16, 17, 19]. For example, Young and De Klerk [17] found alcohol prevalence rates of almost 50% at Rhodes University. At the same South African university, 2 years later, Young and Mayson [19] found that 57.9% of the sample reported hazardous alcohol consumption, i.e., four or more drinks at a time on at least three separate days in the previous three months.

Similarly, another South African study carried out by Kyei and Ramagona [15], at the University of Venda, found that while over 65% of the students surveyed *use* alcohol, 49% of those students *abuse* it. A more recent study conducted by Du Preez and colleagues [2], which focused on the drinking behaviour of students at Stellenbosch University reported that 71% of males and 54% of females reported hazardous drinking patterns. In addition, the study also found that 13% of the sample indicated harmful drinking behaviour.

The concern of such findings lies in the potential short- and long-term adverse effects associated with the use of substances on students’ overall health and well-being. As substance use has been associated with an increased risk of contracting communicable diseases such as HIV/AIDS and TB [20]; non-communicable diseases such as mental illnesses; maternal and child maternal and child morbidity and mortality [21]; as well as injury and trauma. The previously noted consequences of substance use and abuse notwithstanding, it has also been recognised to contribute to epidemics of crime and violence, high university dropout rates, unemployment, and high levels of poverty and crime [22].

Research has shown that to intervene effectively and prevent the negative consequences of substance use, it is important to identify socio-demographic [18, 23, 24], environmental and psychological factors [25–27] contributing to the use and misuse of substances. According to Becker et al. [25] and NIDA [27], mental conditions such as stress, anxiety, and depression are important factors predisposing students to use and subsequently abuse substances [25, 26]. The present study, therefore, aimed to establish the prevalence and associated factors of substance use among students at a historically disadvantaged university in the Western Cape, South Africa.

The study attempted to answer the following questions: (1) What is the prevalence rate of substance use amongst students at the University? (2) What types of substances do the students commonly use? (3) What are the factors associated with substance use among university students?

## Method

### Design and context

This quantitative study employed convenience sampling to recruit university students who were 18 years and older from one of 26 public universities situated in South Africa. The university consists of seven faculties and four schools. Faculties are made up of Arts, Community and Health Sciences, Dentistry, Economic and Management Sciences, Education, Law and Natural Science. The schools include Pharmacy, Government, Nursing and Science and Mathematics. The study was primarily borne out of the realisation that very little was known about the current prevalence and factors associated with substance use and abuse at universities in the Western Cape, even though it is situated in the region where alcohol and drug use is reported to be four times higher than the national average in South Africa [28].

### Procedure and ethics

The study was approved by the university and its ethics committee (BM18/9/1). After receiving a list of all registered students (N = 25,226) from the Registrar of the university, we sent out emails (as well as reminders) to all the student email addresses. The email included the description of the study and the link to the questionnaire. The questionnaire was administered online using SurveyMonkey for a period of two months (31 July to 30 September 2019). A link to the questionnaire was sent to students' university email addresses. Upon accessing the questionnaire, participants were provided with an information sheet and consent form which outlined the purpose, aims and, objectives of this study, the rights and responsibilities of the participants, as well as what it is that would be expected from them should they agree to take part in the research. Through the information sheet and consent forms, participants were also assured that their identity would remain confidential, and their responses used for research purposes only. This was ensured by not requiring any identifiable information from participants, thus maintaining their anonymity. The participants in this study were provided with referral pathways, should the need for counselling services or emergency intervention arise as a result of their participation in this study. Should students have required any additional referrals for social or mental health support, they were provided with telephone and email contact details of possible referrals. The researcher’s contact information was also available should they not have been successful on their own.

### Participants

After excluding incomplete and missing data, 2915 questionnaires were deemed valid for analysis (11.6% response rate). Participants with two missing values on either the AUDIT or the DUDIT as well as corresponding missing values for their demographic information, which would have resulted in biased calculations, were removed. See Table 1 for the demographic characteristics of the population under study.

**Table 1:** Demographic characteristics from the student sample (n = 2915)

	Count	%
*Age*		
18–24	2164	74.2
25–34	495	17.0
35–44	172	5.9
45–54	68	2.3
55–64	6	0.2
65–74	2	0.1
≥ 75	0	0.0
Missing	8	0.3
Total	2915	100.0
*Gender*		
Female	1863	63.9
Male	990	34.0
Non-binary/third gender	15	0.5
I prefer not to answer	25	0.9
I prefer to self-describe	8	0.3
Missing	14	0.5
Total	2915	100.0
*Relationship status?*		
Single	1571	53.9
In a relationship	1054	36.2
Married	255	8.7
Widowed	2	0.1
Divorced	13	0.4
Separated	9	0.3
Missing	11	0.4
Total	2915	100.0
*Year of study*		
1st year	839	28.8
2nd year	748	25.7
3rd year	667	22.9
Honours	327	11.2
Masters	240	8.2
PhD	66	2.3
Missing	28	1.0
Total	2915	100.0
*Faculty of registration*		
Arts and Humanities	646	22.2
Community and Health Sciences	299	10.3
Law	146	5.0
Education	206	7.1
Natural Science	415	14.2
Dentistry	72	2.5
Economic and Management Sciences	1013	34.8
School of Nursing	38	1.3
School of Pharmacy	26	0.9
School of Government	44	1.5
School of Science and Mathematics Education	4	0.1
Missing	6	0.2
Total	2915	100.0
*Residence*		
University on-campus residence	387	13.3
University off-campus residence	248	8.5
Living at home with parents/family	1517	52.0
Private accommodation	745	25.6
Missing	18	0.6
Total	2915	100.0
*Are you originally from the Western Cape?*		
Yes	1735	59.5
No, I moved here to attend university	521	17.9
No, my family relocated	82	2.8
If "no", where are you originally from?	566	19.4
Missing	11	0.4
Total	2915	100.0

The final sample consisted of 34% men, 64% women and 1.7% who presented as “other” in terms of ‘gender’. Participants’ ages were captured categorically, ranging from 18–24 years to 75 years and older. The majority of the sample fell into the two youngest categories, i.e. [18–24 years (n = 2164 (63.9%)] and 25–34-year-old category (17%) respectively. With reference to the sample’s level of study, a large proportion of the participants were 1st year (28.8%), 2nd year (25.7%) and 3rd year (22.9%), undergraduate students. Most of the sample (34.8%) was from the faculty of Economic and Management Sciences. This statistic was succeeded by the second largest grouping 22% of students in the faculty of Arts and Humanities.

### Measures

The substance use questionnaire consisted of five different instruments namely, the demographic section (please see Additional file 1 for a copy of the demographic section of this questionnaire), the Alcohol Use Disorders Identification Test [29], The Drug Use Disorders Identification Test [30], The Perceived Stress Scale [31] and The Self-Reporting Questionnaire [32].

#### Demographic section

A demographic section was developed in order to ascertain demographic information relevant to the current study’s aims and objectives. Questions regarding the students’ substance use, age, gender, education level, year level, marital status and onset of substance.

#### The Alcohol Use Disorders Identification Test (AUDIT)

The Alcohol Use Disorders Identification Test (AUDIT) was employed to help screen, categorise and diagnose the incidence of safe, hazardous, harmful and dependent drinking among students. The AUDIT is a brief 10-item, 5-point Linkert scale, self-administered questionnaire, with responses ranging from 0 (never) to 4 (4 + times per week) [33]. The AUDIT has demonstrated a high degree of internal consistency, yielding a Cronbach’s Alpha score of 0.83, with a range of 0.75–0.97 [34]. For the current sample, Cronbach’s alpha was 0.82.

#### The Drug Use Disorders Identification Test (DUDIT)

The DUDIT was employed in order to determine the extent of drug use among students. The DUDIT was developed to assist in the screening, diagnosing and categorising the severity of use of substances other than alcohol [30]. This self-report questionnaire uses a 5-point Likert scale which categorises individuals into three broad categories of drug use, namely, “no drug related problems”, “harmful use or dependence” and “heavily dependent on drugs”. The DUDIT was found to be a psychometrically sound instrument with high convergent validity (r = 0.85) when compared to 44 similar measures such as the DAST-10 and has a Cronbach's alpha of 0.94 [35]. For the current sample, the Cronbach’s alpha was 0.88.

#### The Perceived Stress Scale (PSS-10)

The PSS-10 is one of the most extensively used instruments for measuring self-perceived stress on a scale from 0 (never) to 4 (very often) [36]. Tallied PSS scores are used to detect three categories of stress. An individual is considered to be experiencing low stress is their respective scores ranges from 0 to 13. Scores ranging from 14–26 suggests moderate stress while scores ranging from 27 to 40 would suggest high perceived stress [31]. The PSS-10 has shown to have good internal and test–retest reliability (α = 0.84–0.86) and it has demonstrated convergent validity with measures of anxiety, depression, and health, and divergent validity with measures of sensations-seeking, religious faith, and aggression among university students [37, 38]. For the current sample, Cronbach’s alpha was 0.58.

#### The Self-Reporting Questionnaire (SRQ-20)

Developed by the World Health Organization (WHO), this questionnaire is a self-rating scale specifically designed to screen for psychological discomfort among individuals, particularly in developing countries. The SRQ-20 was therefore employed to assess the frequency and severity of 20 symptoms related to depression and anxiety among students. The SRQ has proven to be a valid (Cronbach’s α = 0.85) [39] and reasonably stable instrument in a several studies in different cultural contexts [40, 41]. Both the PSS-10 and SRQ-20 are two of the most widely used instrument to measure perceived stress [36–38] and psychological distress among populations in several different cultural contexts in and around South Africa [40, 41]. For the current sample, Cronbach’s alpha was 0.89.

### Data analysis

Data were entered into an Excel spreadsheet, and analysis was conducted using the IBM Statistical Package for Social Sciences Version SPSS 26.0 software. Percentages and frequencies were used to report categorical variables. Descriptive statistics was used to summarise the participants’ socio-demographic characteristics and bivariate analysis to examine the associations between background characteristics and alcohol and drug use. The Chi-square test for independence (using α = 0.05) was used to determine whether there were significant differences between student’s substance use before and after their university enrolment. A chi-square test is commonly used when analysing two categorical variables from a single population [42]. Because scores were not normally distributed, Mann–Whitney U-tests were used to determine the association between students’ self-reported mental health and their use of substances. A *p* value of less than 0.05 was determined to be statistically significant.

## Results

### Prevalence of substance use amongst students

The prevalence rates of substance use among the sampled students are presented in the table below (Table 2). The prevalence results presented were based on the results obtained questions in the demographic questionnaire. For students to have been labelled as a “substance user” students had to have responded ‘yes’ to the question, which read, “Are you still using any of the substances mentioned above?”. In order to be considered a “non-substance user”, students had to have indicated ‘no’ substance use with respect to this question. Respondents were labelled “unsure” if they indicated ‘yes’ to this question but had *not* selected any of the substances listed in the question which followed on the survey.

**Table 2:** Substance use while at university

	Frequency	Percent
Substance **user**	1827	62.7
**Non**-substance user	1084	37.2
Unsure	4	0.1
Total	2915	100.0

The table above reports on the prevalence of the sampled students who indicated that they had used any one of the substances listed in the demographic questionnaire. Confidence interval: 62.68% [95% CI 60.89, 64.43]

The findings presented in Table 2 show that the majority of respondents reported using substances after they enrolled at the university (62.7%). In this dataset, chi-square analysis indicated a narrow interval span of 60.89–64.43% can be observed among students with an odds ratio of 0.5, and a 95% confidence level. This is indicative of the chances of using substances after being exposed to the university environment is 50%.

### Types of substances used

Table 3 (below) displays a list of the substances reported to have been used by the participants after their enrolment at university. Alcohol was the most used substance among students (80.6%) (*n* = 1472). The second-most used substance reported by respondents is cannabis, which is commonly known in the Western Cape as ‘dagga’ or weed. The percentage of students reporting cannabis use amounts to 46% (*n* = 841) of the sampled respondents. The third largest proportion of students (96 students) indicated that they used ecstasy.

**Table 3:** Reported substances used

	Total	Percent
Prescription or non-prescription medication	85	3.18
Alcohol	1462	54.72
Cannabis	837	31.32
Methamphetamine	13	0.49
Buttons (Mandrax)	5	0.19
Unga (heroin-based drug)	1	0.04
Ecstasy	96	3.59
Other	146	5.46
Missing	27	1.01
Total	2672	100.00

Table 3 further reveals that 8% of the students reported using substances that were not explicitly listed in the questionnaire. These substances were listed by participants as: Ritalin (*n* = 33), Poppers (*n* = 32), Ketamine (*n* = 31), Mescaline (*n* = 30), other over-the-counter-medicine (*n* = 28), Dimethyltryptamine (DMT or N) (*n* = 27), Xanax (*n* = 26), Vape (*n* = 25) Traditional beer (*n* = 24), Pethidine (*n* = 21) Tramadol (*n* = 20), Rock (*n* = 19), Hookah (*n* = 18), Flakka (*n* = 17), CAT (*n* = 16) MD (n = 15), MDMA (*n* = 14), Acid (*n* = 10) LSD (*n* = 12) Mushrooms (*n* = 13) and Cocaine (*n* = 11).

#### Level of Alcohol and Drug Use among University Students (AUDIT and DUDIT)

The AUDIT and DUDIT were used to determine the amount of alcohol and drug use among the sample of students who stated that they were current substance users in order to supplement the results from the prevalence findings presented above. The results of the Alcohol Use Disorder Identification Test (AUDIT) and the Drug Use Disorder Identification Test (DUDIT) are presented in Table 4 below. In order to determine the extent of substance use by those who identified as using substances, the analysis was conducted on the 1827 participants who reported using substances after enrolling at university.

**Table 4:** Substance users’ level of alcohol and drug use

	Total	Percentage
AUDIT categories		
Low-risk drinking	819	70.4
Hazardous drinking	269	23.1
Harmful drinking	37	3.2
Alcohol dependence	39	3.4
Total AUDIT	1164	100
DUDIT categories		
No drug-related problems	1248	87.2
Harmful use or dependence	172	12.0
Heavily dependent	12	0.8
Total DUDIT	1432	100

A total of 1164 (63.7%) of the 1827 students labelled as ‘substance users’ completed the AUDIT. Although the majority of that cohort indicated ‘low-risk drinking’, a total of 349 students indicated ‘hazardous’, ‘harmful’ drinking patterns and ‘alcohol dependence’.

Most respondents (87.2%) who completed the DUDIT reported, what the DUDIT defines as, ‘no drug-related problems’ while 184 students reported ‘harmful/dependence’ and ‘heavily dependent’ use.

### Factors associated with substance use

#### Self-Report Questionnaire-20

A cut off score of 7/8 is used to indicate the presence of depression and anxiety [41] or as Harpham et al. [43] reports, ‘mental ill health’. Thomas [44] used a cut-off score of 7/8’s in a study in Durban, South Africa. The table below (Table 5) indicates that 32.5% of the total sample, or 45.1% who completed the SRQ-20 presented with a possible case of mental ill health. This indicates the prevalence of mental health distress as reported within this sample.

**Table 5:** Self-report questionnaire 20

	Frequency	Percent
Valid		
None/non-case	1152	54.9
Indicated/case	948	45.1
Total	2100	100.0
Missing		
System	815	
Total	2915	

A Mann–Whitney U Test was conducted in order to test the significance of the association between those who used substances, those who did not and their associated SRQ score. The results are presented in Table 6 below.

**Table 6:** SRQ-25 scores across substance users and non-substance users independent-samples Mann–Whitney U test

Summary	
Total N	2097
Mann–Whitney U	467,533.000
Wilcoxon W	767,458.000
Test statistic	467,533.000
Standard error	13,356.488
Standardized test statistic	− 3.329
Asymptotic Sig. (2-sided test)	**0.001**

**Fig. 1:**
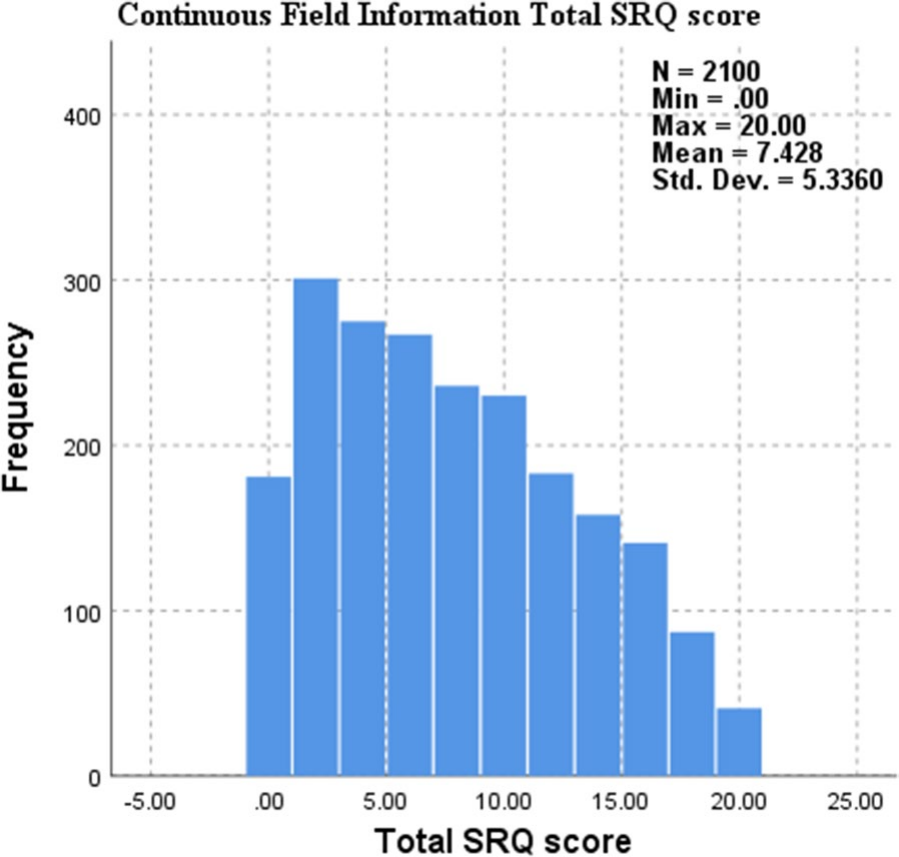
SRQ-25 × Substance Use Histogram

**Fig. 2:**
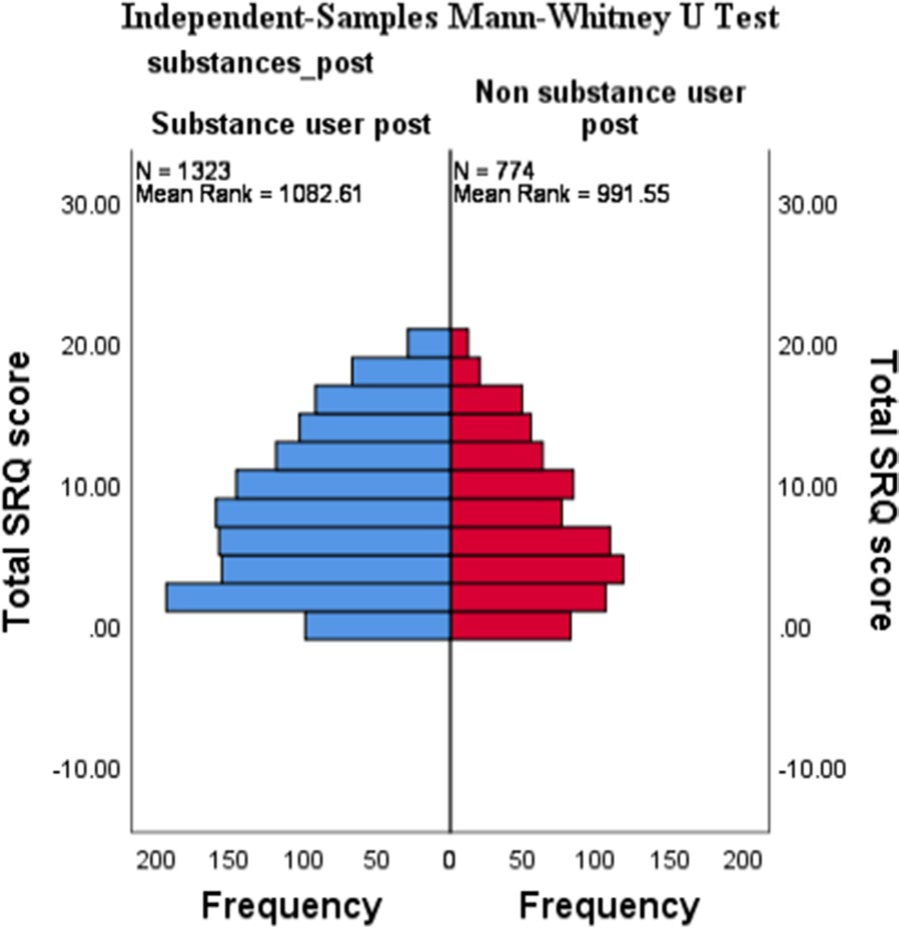
SRQ-25 ×Substance Use Independent-Samples Mann-Whitney U test

The results found in Table 6 and Figs. 1 and 2 reveals a significant association (*p* < 0.01) between being a substance user and non-substance user and students’ respective SRQ scores. The results indicated that students who scored higher in the SRQ-20 (possibly indicating depression and anxiety symptoms) were students who reported substance use at university.

A further cross tabulation presented in Table 7 above highlights that those students who reported hazardous, harmful drinking and alcohol dependence also report higher levels of mental health concerns (SRQ-20). Table 8, below, also indicates that those who report hazardous, harmful drinking and alcohol dependence also report higher levels of perceived stress as compared to those with lower levels of stress. It is interesting to note that all everyone who reported moderate to high perceived stress participated in hazardous or harmful drinking. This speaks to other protective factors which might buffer alcohol use.

**Table 7:** Cross tabulation of AUDIT categories with self-report questionnaire

	SRQ-20		Total
	Not indicated	Indicated	
AUDIT_Categories			
Low risk or safe drinking	616	448	1064
Hazardous drinking	154	168	322
Harmful drinking	12	35	47
Alcohol dependent	13	28	41
Total	795	679	1474

**Table 8:** Cross tabulation of AUDIT categories with Perceived Stressed Scale

	PSS_Categories			Total
	Low perceived stress	Moderate perceived stress	High perceived stress	
AUDIT categories				
Low risk or safe drinking	72	1002	69	1143
Hazardous drinking	8	289	41	338
Harmful drinking	0	43	6	49
Alcohol dependent	2	38	5	45
Total	82	1372	121	1575

### Perceived stress scale-10 results

To ascertain the levels of stress students’ experience during their time at university, the PSS-10 was administered and analysed. The PSS-10 measures the level at which respondents appraise life events as being unpredictable, overwhelming, or challenging. Individual scores on the PSS range from 0 to 40, with higher scores indicating higher perceived stress between 0 and 13 are perceived to have low stress. Scores ranging from 14 to 26 would indicate those whose scores are considered as having moderate stress. The final category of scores, i.e. ranging from 27 to 40 would be indicative of individuals having high perceived stress. It is within this context that respondents were scored, and findings were analysed.

Table 9 indicates that substance users have a higher level of perceived stress versus those who do not use substances. The table below indicates whether this difference was significant using a Mann–Whitney U Test (Table 10).

**Table 9:** Cross tabulation of substance user and non-user versus perceived stress

	PSS_Categories			Total
	Low perceived stress	Moderate perceived stress	High perceived stress	
Substance user post university				
Non-substance user	52	711	55	818
Substance user	84	1220	108	1412
Total	136	1931	163	2230

**Table 10:** PSS-10 results: independent-samples Mann–Whitney U test summary on users and non-users

Total N	2230
Mann–Whitney U	560,076.000
Wilcoxon W	895,047.000
Test statistic	560,076.000
Standard error	14,617.221
Standardized test statistic	− 1.193
Asymptotic Sig. (2-sided test)	.233

**Fig. 3:**
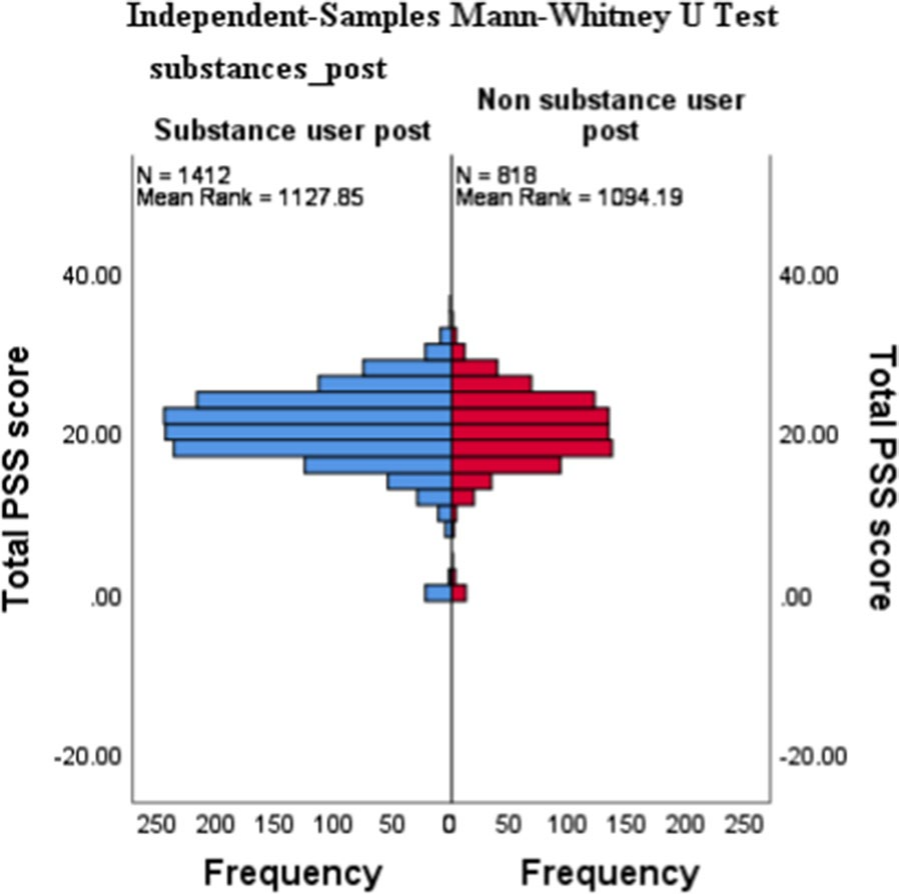
PSS-10 × Substance Use Independent-Samples Mann-Whitney U test

**Fig. 4:**
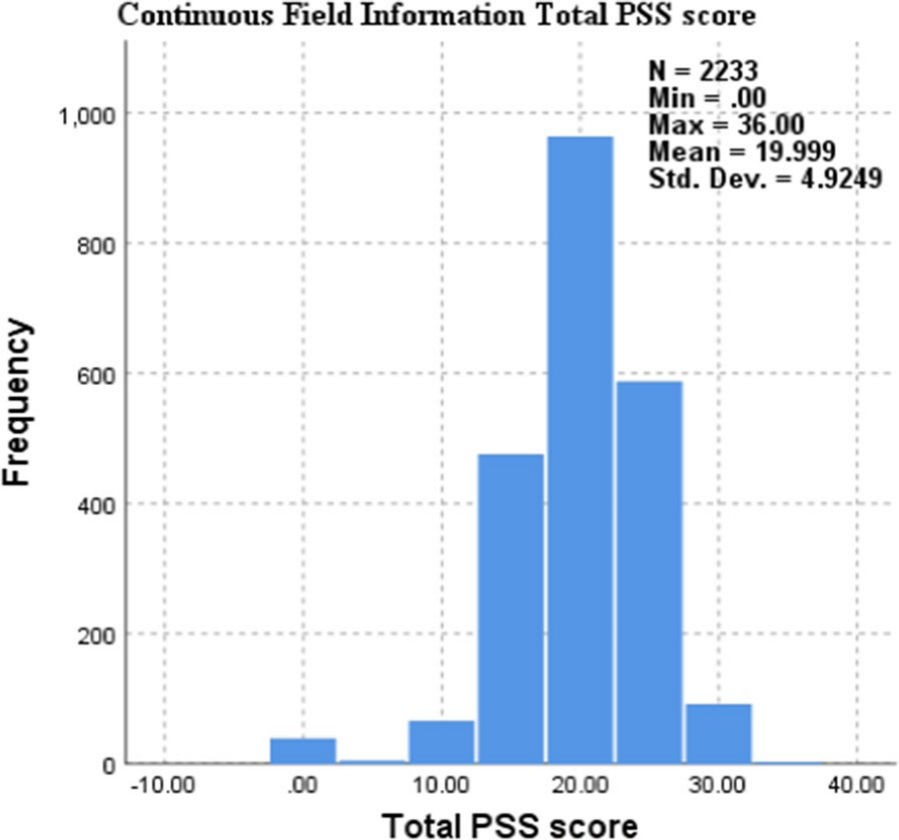
PSS-10 × Substance Use Histogram

The results in Table 8 above and Figs. 3 and 4 below show that there is no significant association (*p* > 0.05) between being a substance user and nonsubstance user post university enrolment and students respective PSS-10 scores. The levels of perceived stress appear to be similar for both cohorts.

Table 11 above, is a cross tabulation of the drug use categories according to the DUDIT and the SRQ. According to the table, those who displayed harmful use or are heavily dependent on substances also report the presence of more mental ill health symptoms. Interestingly, 749 of the 1744 students who reported mental ill health challenges did not indicate drug-related problems.

**Table 11:** Cross-tabulation of DUDIT categories and SRQ-20

	SRQ-20		Total
	Not indicated	Indicated	
DUDIT_Categories			
No drug-related problems	995	749	1744
Harmful use or dependence	91	123	214
Heavily dependent	0	14	14
Total	1086	886	1972

A similar cross tabulation (Table 12) was conducted on the DUDIT categories and students’ perceived levels of stress. Although many students indicated a moderate to high level of perceived stress (n = 1608 and 125 respectively), these students did not report problems with drug use. However, a larger number of students reported moderate to high perceived stress who were also using substances in a harmful or dependent manner.

**Table 12:** Cross-tabulation of DUDIT categories and PSS

	PSS			Total
	Low perceived stress	Moderate perceived stress	High perceived stress	
DUDIT_Categories				
No drug-related problems	117	1608	125	1850
Harmful use or dependence	7	194	23	224
Heavily dependent	0	9	5	14
Total	124	1811	153	2088

## Discussion

### Prevalence of substance use

The aim of this study was to determine the prevalence of, or patterns, of substance abuse among university students as well as mental health factors that may be influencing this use. The term substances refer to both alcohol and drug use. Results from the study show that 62.7% of sampled students indicated, not only that they used substances but that this use (both alcohol and other substances) started *after* enrolling at the university. The prevalence rates found in this study appear to align with key findings which, similarly, found high substance use rates after students had enrolled at their respective university/college [1, 4, 10–12].

The extent of alcohol and drug use was evaluated using the AUDIT and DUDIT. These results are particularly interesting as it revealed that the majority of students in the sample reported ‘low-risk drinking' (70.4%) and ‘no drug-related problems’ (87.2%). These results are in direct contrast with similar studies in the field which report relatively high prevalence of substance abuse among university students, particularly in South Africa [2, 15, 19]. The substance use rates reported could be due to a variety of reasons, one of which concerns social desirability bias, which refers to the tendency respondents have to present and align themselves and their reality with what they believe to be socially acceptable. However, each questionnaire was self-report and could be completed alone, without the presence of an interviewer or someone known to the student. The majority use notwithstanding, both the AUDIT and DUDIT revealed a number of students who should be considered for either brief or intensive interventions for substance use.

### Types of substances used by students

The three most commonly used substances reported were alcohol (80.6%), cannabis (46%) and ecstasy (5.3%) amongst those who used substances after university enrolment. Interestingly, ecstasy use was found to be higher than methamphetamine use among students in the Western Cape, an area well-known for its increased methamphetamine use [45]. “Other” substances accounted for 8% of the sample, important to note here is that the number of “other” substances evidently surpasses commonly well-known and well-documented substances such as ecstasy (n = 96), methamphetamine (n = 14), buttons (n = 6) and unga (n = 1). This noteworthy and rather unanticipated finding could be indicative of a *shift* in the types of substances commonly reported/used by university students at this point in time. Although the sample is not representative, this finding is important as it provides an opportunity for researchers and healthcare practitioners to be mindful of the types of substances being used by individuals of a certain age range in the Western Cape.

### Mental health of students

The results of this study revealed a significant association (*p* < 0.01) between students’ substance use and their respective SRQ scores. These results appear to be in consensus with much of the available literature which interchangeably associates some aspect of mental health with substance among university students [25, 27, 46]. However, although valuable, the results presented are likewise unable to support or refute the three main hypotheses offered in the literature review which varied from conclusions relating to whether substance use leads to mental health problems [47]; whether mental health problems cause substance [48] or whether these concepts are so closely related that it could not be studied in isolation [49]. It is likewise unfortunate that results were unable to answer the question as to whether students who already present mental health problems prior to attending universities are at an even higher risk of experimenting with substances, as the new environment could exacerbate their already existing symptoms [50]. What it does however show, is that there appears to be a significant difference between the two groups in terms of their mental health and use of substances, i.e., students who identified as non-users reported fewer symptoms of anxiety and depression (according to the SRQ-20) than students who indicated that they were substances users. Which is indicative of there being some validity to the claims concerning the complex relationship between substance use and mental health, particularly among university students.

This study used the PSS-10 to measure psychological stress among students mainly because it defines stress as an interaction between environmental demands and the individual’s capacity to cope [31]. Results demonstrate that there is no significant association (*p* > 0.05) between being a substance user and a nonsubstance user post university enrolment and students' respective PSS-10 scores. Figures 1 and 2 furthermore support and expand on the results found in Table 9 by showing how scores cluster around the 19/20 mark in both groups. In terms of students’ level of stress, the results from Figs. 1 and 2 furthermore indicate that the majority of students in both groups fall within the moderate stress levels as per PSS-10. These results found above are in contrast with a body of literature that found significant associations between respondents who suffered from psychological distress and their use of substance use. Substance use was commonly reported as being used by distressed respondents to cope with academic pressures and demands [50, 51]. Interestingly, even though we did not account for risk and protective factors, based on the results of the AUDIT/DUDIT category cross tabulation with the SRQ and PSS, protective factors could be moderating or mediating the relationship between stress/mental ill health and students’ either using substances or not. Such factors could account for those with high stress and mental health challenges and not having a substance use problem per se. Considering the scarcity of knowledge about the risk and protective factors of substance abuse among university students, a follow-up study to investigate such factors should be prioritised.

### Implications of the study

The insights to be gained from this study could serve several purposes and contribute towards the prevention and reduction of substance use and/or abuse among university students in several ways. In its entirety, the study contributes to the overall scarcity of existing knowledge on substance use and abuse among university students in South Africa. The study sheds light on the current prevalence and the extent to which students’ use and/or abuse substances in a previously underexplored population in the Western Cape. Although it does show that the large majority do not necessarily have harmful or hazardous substance use patterns, it is noteworthy that a minimum of 184 to a maximum of 359 students reported harmful/hazardous/dependent use. Therefore, awareness campaigns and varying degrees of referrals and interventions should be made available to students.

In addition to reporting on the “conventionally” well-documented substances, such as alcohol, marijuana, methamphetamine and heroin in South Africa, this study also provided a platform where students could disclose their use and/or abuse of other types of substance use. This information could prove useful for any future attempts to tailor, inform and/or contextualise research endeavours of a similar nature. The unanticipated findings relating to the assortment of substances could be indicative of a gradual paradigm shift in the types of substances commonly reported/used by students. The novel findings of this study could serve as a baseline input to inform policy makers, programme developers, service providers, parents, and other stakeholders who are involved in the design and implementation of more effective awareness, prevention and needs-based intervention services; and the findings of this study could also serve as a feature map for future research relating to substance use in and around South Africa.

## Conclusions

### Limitations

The results produced in this research study, although valuable, reflects a single, purposefully selected university in the Western Cape. Since the prevalence and nature of substance abuse among university students in the Western Cape may vary depending on the environments where the universities are found, the generalisation of the current study’s findings should be done with caution. A general limitation of a correlational study is that it can determine the association between variables but cannot predict causation. Another limitation of inherent is such studies is the ability of respondents to accurately recall past events. In this case, the time intervals for which respondents were asked to report their substance use were not specific, i.e., no reference periods were used to restrict and specify the time intervals for which respondents reported their use of substances. This oversight may have produced unclear assumptions regarding the prevalence of substances among students. In addition, tobacco use was not classified as a problematic substance in this study. This is perhaps something to consider in future studies of this nature.

It is also essential to mention here that the questionnaire was disseminated in only one of the three official languages within the Western Cape, i.e., English. This was done in order to align with the University’s primary medium of teaching and of examination, which is, English. This being said, the official language policy of the Western Cape Government (Western Cape Government 2019) encourages the promotion and use of all of the three official languages of the Western Cape, namely Afrikaans, isiXhosa and English where possible. It is thus recommended that future research carried out in different provinces and or countries consider the official languages of the population under study and strive to provide the respondent with an equal opportunity to interpret and answer questions in their mother tongues.

The social desirability bias, inherent in the self-report measures on substance use, may have resulted in students providing socially acceptable rather than honest answers. Apart from the social desirability bias, it is important to bear in mind that students received the online link via their university student email addresses within a specific timeframe, from 29 July to 27 September 2019. The implications here are that students who were absent, or who did not have access to the necessary resource to complete the questionnaire could have been excluded. Although useful, the cross-sectional design has been criticised for only examining aspects of individual’s beliefs and behaviours without paying concerted consideration to the context in which these beliefs and behaviours occur, which could account for misinterpretation of meanings of the beliefs and/or behaviour recorded. As such, it is necessary to exercise caution when interpreting the results of this study.

### Recommendations of the study

Taking the aforesaid findings into consideration, it is hoped that the current study’s results would call upon researchers to further investigate the association of factors in relation to an array of substances other than alcohol. This might be essential in the identification of an increase and/or decrease of many substances, and the identification of newly introduced substances, which is vital for the creatiion of awareness-, and prevention- campaigns as well as intervention strategies aimed at the population under study.

A conclusion of the limitations and recommendations of this study cannot be complete without calling for more comprehensive efforts (multidisciplinary) when investigating the use and abuse amongst individuals in our society. Since this research is observational, experimental research is recommended to identify effective intervention options for mitigating the burden of substance abuse among university students.

Discovering these influencing factors (both risk- and protective) would decrease the probability of an individual using drugs, and would once again pinpoint areas one could focus on with interventions, which would empower those in need of intervention, as opposed to educating only. A determination should also be done on evidence-based “best practices” for primary prevention, as well as the treatment of substance abuse among adolescents in South Africa.

In attempts to promote and adhere to the ethicical principles of avoiding harm, giving respect and protecting participants’ integrity, the author's advocacy plea is to cultivate more inclusivity in future research endeavours, especially in the social sciences. Such efforts could begin by exploring social constructs such as gender on a non-binary spectrum. In addition to this, more culturally sensitive, multi-wave longitudinal research needs to be carried out in order to improve on and expand the understanding of substance use and abuse among young people in South Africa, particularly those transitioning from childhood to adolescence to young adulthood, during which pervasive individual and contextual change is the bedrock of these developmental transitions.

## Conclusion

The overall aim of the study was to explore the prevalence and factors associated with substance among university students in South Africa in order to provide baseline information that could inform the development and/or tailoring of any awareness and or prevention campaigns designed to reduce substance use and abuse among students in South Africa. Perhaps more importantly, it is hoped that the results, implications, limitation, and recommendation of the present study invokes increased focus and ignites novel or innovative thinking when undertaking research of similar nature.

## Supplementary Information

**Additional file 1**. Demographic Section. A demographic section was developed in order to ascertain demographic information relevant to the current study’s aims and objectives. Questions regarding the students’ substance use, age, gender, education level, year level, marital status and onset of substance.

## Data Availability

The datasets used and/or analysed during the current study are available from the corresponding author on reasonable request.

## Abbreviations

α: Alpha (level of significance)
N: Equals the size of the population
n: Equals the sample size
p: *p* value
