# Supplementary material for: Prevalence and factors associated with substance use among university students in South Africa: implications for prevention
Source: BMC Psychol. 2022 Dec 15;10:309. doi: 10.1186/s40359-022-00987-2 (PMC9753402; doi:10.1186/s40359-022-00987-2)
Supplement: Supplementary file 1 — Additional file 1. Demographic Section. A demographic section was developed in order to ascertain demographic information relevant to the current study’s aims and objectives. Questions regarding the students’ substance use, age, gender, education level, year level, marital status and onset of substance. [file 40359_2022_987_MOESM1_ESM.pdf]

Substance Use Questionnaire

Demographic Information

UNIVERSITY of the WESTERN CAPE

UNIVERSITY OF THE WESTERN CAPE
Private Bag X 17, Bellville 7535, South Africa
Tel: +27 21-9392283 Fax: +27 21-959 3515

DEMOGRAPHIC INFORMATION

Please complete the following and where appropriate tick the applicable answer.

1. What is your age?

   ○ 18 to 24
   ○ 25 to 34
   ○ 35 to 44
   ○ 45 to 54
   ○ 55 to 64
   ○ 65 to 74
   ○ 75 or older

2. Please tick the gender that best represents you

   ○ Female
   ○ Male
   ○ Non-binary/third gender
   ○ I prefer not to answer
   ○ I prefer to self-describe

3. What is your current relationship status?

   ○ Single
   ○ In a relationship
   ○ Married
   ○ Widowed
   ○ Divorced
   ○ Separated

4. Year of study

- ◯ 1st year
- ◯ Honours
- ◯ 2nd year
- ◯ Masters
- ◯ 3rd year
- ◯ PhD

5. Faculty of registration

- ◯ Arts
- ◯ Economic and Management Sciences
- ◯ Community and Health Sciences
- ◯ Natural Science
- ◯ Law
- ◯ School of Nursing
- ◯ Education
- ◯ School of Pharmacy
- ◯ Natural Science
- ◯ School of Government
- ◯ Dentistry
- ◯ School of Science and Mathematics Education

6. Residence

- ◯ University on-campus residence
- ◯ University off-campus residence
- ◯ Living at home with parents/family
- ◯ Private accommodation

7. Are you originally from the Western Cape?

- ◯ Yes
- ◯ No, I moved here to attend university
- ◯ No, my family relocated
- ◯ If "no", where are you originally

____

8. Have you ever used any of the following substances before attending university?

- ◯ Alcohol
- ◯ E (Ecstasy)
- ◯ Cannabis (Dagga)
- ◯ Tobacco substances (cigarettes, chewing tobacco, cigars, e-cigarettes, etc.)
- ◯ Methamphetamine (Tik)
- ◯ None
- ◯ Buttons (Mandrax)
- ◯ Unga (Heroin)
- ◯ Other (please specify)

9. Have you ever abused any prescription or non-prescription medication (sedatives, stimulants, painkillers etc) before attending university?
   - ◯ Yes
   - ◯ No
   - ◯ If “yes” which medication(s)

10. Have you ever abused any prescription or non-prescription medication after starting university?
    - ◯ Yes
    - ◯ No

11. Which of the following substances have you used after enrolling at the university?
    - ◯ Alcohol
    - ◯ Cannabis (Dagga)
    - ◯ Methamphetamine (Tik)
    - ◯ Buttons (Mandrax)
    - ◯ Unga (Heroin)
    - ◯ Other (please specify)
    - ◯ E (Ecstasy)
    - ◯ Tobacco substances (cigarettes, chewing tobacco, cigars, e-cigarettes, etc.)
    - ◯ None

12. Are you still using any of the substances mentioned above?
    - ◯ Yes
    - ◯ No
    - ◯ If "yes", which substances(s)?

13. Have you ever used any type of substance on the university premises?
    - ◯ Yes
    - ◯ No

◯ If "yes", which substances(s)?

14. Has your substance use increased since starting university?

◯ Yes

◯ No
